# The American Dental Society of Europe

**Published:** 1890-10

**Authors:** 


### THE AMERICAN DENTAL SOCIETY OF EUROPE.—SEVENTEENTH ANNUAL MEETING, PARIS, AUGUST 6 AND 7, 1889.

(Continued from page 481.)

### *First Day.—Afternoon Session.*

Dr. Wilhelm Sachs read his paper on "Various Modes adopted in filling Teeth" (see page 600), followed by Dr. Bryan's paper on "Improved Dental Ledger and Book-Keeping" (see page 606).

### DISCUSSION ON DR. SACHS'S PAPER.

*Dr. Mitchell.*—I think the greatest amount of praise is due to Dr. Sachs for the interest he has taken in the society. There is nothing that can be said in regard to the paper, the work speaks for itself.

*Dr. Bryan.*—There is one point which Dr. Sachs has overlooked, in regard to this paper, and that is the classification of the different teeth. On the last page, No. 1 represents a central incisor right side upper jaw, and he indicates the right and left upper and lower teeth very graphically and very quickly; that is a point in recording our operations of which we can take advantage, saving a great amount of space.

### DISCUSSION ON DR. BRYAN'S PAPER.

*Dr. Mitchell.*—From the fact that all the surfaces of the teeth are exposed here, I think it a decided step in advance. It was not quite clear whether the operations or the teeth were numbered.

*Dr. Rosenthal.*—Mr. President, it seems to me that each operation should be numbered; for instance, if you have a tooth to be treated, three, four, or five times, it should be numbered at each time, and all you need to do is to refer to those numbers on your diagram, and you have the whole history recorded for years and years for reference, when the patient comes.

*Dr. Elliott.*—Of course, any system to have a general adoption must be exceedingly simple and brief. According to my observation, the average dentist is a lazy man: many keep only one or two books, others half a dozen.

*Dr. Rosenthal.*—There are no deciduous teeth on your chart. Have you not sometimes milk teeth to which you want to refer?

*Dr. Bryan.*—I don't keep a record of operations on temporary teeth; but I would say that Dr. Rosenthal is quite correct with regard to reporting the operations. We treat a tooth, fill it temporarily, and by the time the patient has this tooth again treated, we record it as, say, No. 16, right opposite No. 1. Then we fill it with cement. This temporary filling is worn out three years afterwards, and we fill it with gold. We refer to our numbers, it may be ten years afterwards, and we see by Nos. 1–16, etc., that it was treated by an antiseptic method; then we find that No. 16 represents that tooth as having been filled with cement, gold, etc.

If one desires a condensed system of notation, he will find, by referring to Dr. Cunningham's valuable work on the subject, that he has all the necessary abbreviations and paraphernalia of a complete system.

*Dr. Spalding.*—My practice has been to discard all diagrams and simply describe my filling as I do it; for instance, if I make a gold filling in a right superior central incisor, I simply put down the initials of the words.

*Dr. Elliott.*—Why not mesial and distal instead of the letter?

*Dr. Spalding.*—Yes, certainly, that might do; but I do not like that, and it saves a great deal of time.

*Dr. Bryan.*—Here you have a pictorial illustration which will last over fifty years for the use of yourself or your successor.

*Dr. Miller.*—I will show you on the black-board how the students at Berlin mark their operations.

*Dr. Spalding.*—Well, no outsider would understand that.

*Dr. Miller.*—There is no need; it is only you who look at your books.

*Dr. Elliott.*—How do you manage when there are two fillings in the same tooth; do you use the same form for each?

*Dr. Spalding.*—I do.

*Dr. Rosenthal.*—If you have a record, you know at once what you have done for years and years.

*Dr. Spalding.*—Very true; but, in a general way, I find my method quite sufficient.

*Dr. Bryan.*—A patient occasionally comes and declares that the filling which you put in recently has come out; you can by showing him your chart easily undeceive this patient. If a patient is with me for fifteen years (and I hope to be practising so long), I shall have every operation on his chart.

*Dr. Elliott.*—There is one point which seems to be entirely ignored, and that is to give us the data for the durability of different filling-materials. I commenced ten years ago a system which was exhibited before the society at Vevay. I mentioned the fact that I tried to have my books self-explanatory, so that my successor could take the books and would be able to understand them very readily. The point of some importance which I aimed at in the diagram was the line giving room for notes. I have found no difficulty in keeping that up for ten years, and next year I purpose publishing a ten years' report, and I hope to get at some facts which may be of value. Of course you know that this kind of thing cannot be thorough; you can readily see that it is a very great labor, and it will probably take a secretary three months to get at the report and prepare data.

(To be continued.)

